# Supplementary material for: Complexome profiling of the Chlamydomonas psb28 mutant reveals TEF5 as an early PSII assembly factor
Source: Plant Cell. 2025 Mar 17;37(6):koaf055. doi: 10.1093/plcell/koaf055 (PMC12164590; doi:10.1093/plcell/koaf055)
Supplement: koaf055_Supplementary_Data [file koaf055_supplementary_data.zip › psb28_interactive.zip!/plots/intensity/qualityPlots/peptides/_Cre03.g144967.t1.1.html]

### Cre03.g144967.t1.1

Trivial name:   
SeqCoverage [median]: 20.60  
Mass: 68.77 kDa  
  
 

| Sequence | MissedCleavage | Mass [Da] | wtIntensity | muIntensity | Score |
| --- | --- | --- | --- | --- | --- |
| AAEQYAADLQK | 0 | 1206.59 | 259.69 | 1037.21 | 80.24 |
| ALAEAEGR | 0 | 815.41 | 1675.38 | 3304.68 | 104.37 |
| ATEGFSAR | 0 | 837.40 | 690.98 | 0.00 | 84.57 |
| EVVRYEEER | 1 | 1207.58 | 441.11 | 884.44 | 38.57 |
| LFDPEALER | 0 | 1088.55 | 2968.37 | 5371.05 | 80.44 |
| LLAAVQAAVYGAPK | 0 | 1370.79 | 0.00 | 3485.61 | 163.94 |
| LQEEASAR | 0 | 902.45 | 1625.26 | 1527.22 | 119.17 |
| LRVEQQIQAER | 1 | 1368.75 | 516.76 | 0.00 | 57.35 |
| MKQYEDELAR | 1 | 1281.60 | 401.14 | 1753.30 | 72.85 |
| QVAASAANTK | 0 | 959.50 | 275.99 | 518.13 | 109.29 |
| RAAEQYAADLQK | 1 | 1362.69 | 0.00 | 600.23 | 51.73 |
| SDAMSEGLR | 0 | 964.43 | 292.56 | 0.00 | 69.81 |
| TSGMDYAIMSGGDVAPLEGK | 0 | 1997.91 | 0.00 | 574.49 | 115.12 |
|  |  |  | 9147.2 | 19056.4 |  |
